# Screening of Worldwide Barley Collection for Drought Tolerance: The Assessment of Various Physiological Measures as the Selection Criteria

**DOI:** 10.3389/fpls.2020.01159

**Published:** 2020-07-29

**Authors:** Kangfeng Cai, Xiaohui Chen, Zhigang Han, Xiaojian Wu, Shuo Zhang, Qi Li, Muhammad Mudassir Nazir, Guoping Zhang, Fanrong Zeng

**Affiliations:** ^1^Institute of Crop Science, Zhejiang University, Hangzhou, China; ^2^Institute of Crop and Nuclear Technology Utilization, Zhejiang Academy of Agricultural Sciences, Hangzhou, China

**Keywords:** barley, drought tolerance, selection criterion, water relation, osmotic adjustment

## Abstract

Drought is a devastating environmental constraint affecting the agronomic production of barley. To facilitate the breeding process, abundant germplasm resources and reliable evaluation systems to identify the true drought-tolerant barley genotypes are needed. In this study, 237 cultivated and 190 wild barley genotypes, originating from 28 countries, were screened for drought tolerance under the conditions of both water deficit and polyethylene glycol (PEG)-simulated drought at seedling stage. Drought stress significantly reduced the plant growth of all barley genotypes, but no significant difference in drought-induced reduction in the performance of barley seedlings was observed under these two drought conditions. Both cultivated and wild barley subspecies displayed considerable genotypic variability in drought tolerance, which underpinned the identification of 18 genotypes contrasting in drought tolerance. A comparative analysis of drought effects on biomass, water relation, photosynthesis, and osmotic adjustment was undertaken using these contrasting barley genotypes, in order to verify the reliability of the screening and to obtain the credible traits as screening criteria of drought tolerance in barley. As expected, the selected drought-tolerant genotypes showed much less reduction in shoot biomass than drought-sensitive ones under water deficit, which was significantly positively correlated with the results of large-scale screening, confirming the reliability of the screening for drought tolerance under two drought conditions in this study. Likewise, the traits of water relation, photosynthetic activity, and osmotic adjustment differed greatly between the contrasting genotypes under water deficit stress, and they were highly correlated to the growth of barley seedlings, suggesting the potential of them to be the selection criteria for drought tolerance. The analysis of the variable importance of these traits in drought tolerance indicated that sap osmolality and relative water content in the youngest fully-expanded leaf are the suitable selection criteria of screening for drought tolerance in barley at seedling stage.

## Introduction

Drought is the most devastating environmental constraint causing much more yield loss than any other abiotic stress (Farooq et al., 2009; Kadam et al., 2014). It occurs virtually in all climate regions, and the drought-prone areas accounted for 16.2–41.2% of arable land worldwide (Wang et al., 2014; Kebede et al., 2019). Additionally, it has been predicted that the drought frequency and severity will increase in presently dry regions due to climate change (Intergovernmental Panel on Climate Change, 2014). Drought affects almost all stages of growth and development during plant life cycle, inducing dramatic decline in photosynthesis, floral abnormalities, spikelet/kernel sterility, grain yield and quality losses (Kadam et al., 2014). Meanwhile, world population is expected to increase from 7.7 billion currently to 9.7 billion in 2050 (United Nations: Department of Economic and Social Affairs - Population Division, 2019), which will seriously challenge the global food security. To overcome the increasing requirement of agricultural production in the future climate scenarios, the most effective and economic approach is to breed cultivars with high drought tolerance. However, due to limited availability of resistance sources, very little progress in breeding drought tolerant crop has been made.

Barley (*Hordeum vulgare*) ranks the fourth largest cereals in terms of production worldwide with multipurpose use as animal feed, human food, and brewing material. It has been documented that drought stress could severely reduce grain yield by 49–87% in barley (Samarah, 2005; Samarah et al., 2009). Breeding drought-tolerant barley cultivars seems the most effective and economic approach to minimize the adverse effects of drought stress on barley yield production. However, the genetic diversity in barley has been drastically narrowed during long-term domestication, especially by the activities of modern breeding and cultivation (Nevo and Chen, 2010). Genetic variation in barley cultivars was much smaller than that in the wild populations, and only 40% of the alleles found in wild barley were present in cultivars (Ellis et al., 2000). Modern barley cultivars are becoming more vulnerable to abiotic and biotic stresses, and their monotonous genetic background is hindering the breeding and improvement of barley varieties (Zhao et al., 2010). As the progenitor of cultivated barley, wild barley (*Hordeum spontaneum*) shows a much wider adaptability to ecological range differing in water availability, temperature, soil type, altitude, and vegetation (Nevo and Chen, 2010). It has been considered in many researches that wild and cultivated barley genotypes demonstrate varying ability to acclimate to drought stress (Gunasekera et al., 1994; Baum et al., 2003; Lakew et al., 2011; Barati et al., 2015; Barati et al., 2017). Therefore, it is promising to identify the drought-tolerant germplasms from wild barley and to integrate elite traits from wild to cultivated barley for drought tolerance.

Evaluating and identifying drought-tolerant genotypes are critical to all studies concerning drought tolerance (Cattivelli et al., 2008). Diverse genetic resources, reliable traits, and accurate phenotyping methods, as well as appropriate growth stages when drought stress may occur are crucial in identifying drought tolerant materials. Morphological and physiological variations under drought stress are the reflection of plant genetic diversity in drought tolerance, and the genotypes with high adaptability to drought stress can be candidate genetic resources to improve drought tolerance in barley varieties (Nevo and Chen, 2010). So far, a series of agronomic, morphological, physiological, and metabolic traits have been extensively used for screening for drought tolerance, including yield formation (Mwadzingeni et al., 2016), leaf and root morphologies (Hargreaves et al., 2009; Lonbani and Arzani, 2011), biomass production (Szira et al., 2008; Zhao et al., 2010), relative water content (Szira et al., 2008; Yadav et al., 2019), stomatal conductance (Munns et al., 2010), photosynthetic parameters (Lawlor and Cornic, 2002), chlorophyll fluorescence (Oukarroum et al., 2007; Su et al., 2015), and the accumulation of amino acids (Mwadzingeni et al., 2016; Yadav et al., 2019). All these traits have been demonstrated to be significantly correlated with drought tolerance in different plant species, and some of them have been used as the selection criteria in drought tolerant barley breeding programs (Sallam et al., 2019). However, which of these traits could be the superior selection criterion to select the most drought tolerant barley genotypes should be carefully considered (Sallam et al., 2019). For instance, leaf fresh matter, leaf dry matter, and relative water content are considered as the basic traits that are widely used in drought experiments in wheat and barley, whereas Hasanuzzaman et al. (2017) found that the variation in these parameters showed no significant correlation with the genotypic difference in drought tolerance in barley. The authors considered that chlorophyll content and fluorescence parameters could be the highly suitable indicators in screening barley germplasm for drought tolerance (Hasanuzzaman et al., 2017). However, there was also a viewpoint that chlorophyll fluorescence is not a suitable indicator of genotypic differences in the growth response to water stress, because this measurement is generally made on a single leaf and could not reflect the performance of the whole plant (Munns et al., 2010). In addition, many of the above researches on barley used very limited genotypes or genetic population lines for screening for drought tolerance, which might compromise the universality of the results and the possibility of extrapolation to the other genotypes (Varshney et al., 2012; Jabbari et al., 2018; Sallam et al., 2019). Therefore, a large-scale screening practice including hundreds or thousands of barley genotypes with diverse origination regions is very necessary to identify the target genotypes contrasting in drought tolerance.

The search for the best medium for growing plants to impose a controlled water deficit has been going on for decades, without a clear resolution (Munns et al., 2010). In most studies on screening for drought tolerance, drought stress is commonly induced by either (1) terminating water irrigation to plants grown in field trials covered with shelter or pots with soil in greenhouse (Hargreaves et al., 2009; Munns et al., 2010) or (2) creating an elevated osmotic potential to the plants grown in gel or hydroponic medium with different osmoticums like polyethylene glycol (PEG) and mannitol (Luo et al., 2015; Feng et al., 2016; Meng et al., 2016). When applying the treatment of water deficit in the field or in the pots with soil, not only soil drying process is hard to maintain at a uniform and constant water potential through the whole soil profile, but also the effect of drought stress on plants may be affected by other factors like erratic weather patterns, soil-borne diseases, soil mineral nutrition, spatial variation, *etc*. (Munns et al., 2010; Sallam et al., 2016; Sallam et al., 2018). Another problem with soil in pots is that they easily become saturated at the bottom (Passioura, 2006), so that often plants in soil with a moderate water deficit perform better growth than those under the control condition (Munns et al., 2010). All these limitations could lead to a reduction in the repeatability in drought severity and consequently the efficiency of phenotypic selection in the work with real soil. On the other hand, it has been recommended that testing drought tolerance using PEG-stimulated osmotic stress under controlled conditions is useful when working in the ﬁeld where many factors are not controlled (Sallam et al., 2018). But, PEG solution is very viscous and limits O_2_ movement to roots so that the roots become O_2_ deficient, which may interfere with ion transport in plant roots (Munns et al., 2010; Dubois and Inzé, 2020). Furthermore, the practicability of using PEG-induced osmotic stress to represent drought stress has been increasingly queried by several researches (Verslues et al., 2006; Skirycz et al., 2010; Claeys et al., 2014; Dubois and Inzé, 2020), because PEG solutions may induce the short-term hyperosmotic shock to plants but not the gradually-emerged long-term low water potential stress as soil drying does. All the above facts suggest that there is no perfect medium to impose the controlled water deficit to plants, all have limitations including pots containing real soil, that is, soil imported from the field. To ensure the accuracy of the screening for drought tolerance, therefore, it is necessary to evaluate the same genotypes using multi methods of drought stress to select the elite genotypes for target traits.

Accordingly, 237 cultivated and 190 wild barley genotypes originating from 28 countries were screened for drought tolerance in this study at seedling stage under the conditions of both PEG-stimulated and water deficit. Based on their performance in terms of relative fresh weight, relative dry weight, and relative water content of shoots, 18 genotypes were identified showing the constant difference in drought tolerance under two drought conditions. Subsequently, the drought tolerance of these genotypes was further verified under water deficit with various physiological traits including fresh weight, dry weight, relative water content, chlorophyll content and fluorescence, stomatal conductance and leaf sap osmolality. The ultimate aims of the present study were 1) to identify the best suitable drought-tolerant or -sensitive cultivated and wild barley germplasms with the constant difference in drought tolerance under different drought conditions; 2) to develop a feasible and reproducible way of imposing the drought stress on plants; and 3) to assess the suitability of various physiological indices as the feasible criteria of barley drought tolerance for high-throughput screening of barley germplasm at seedling stage.

## Materials and Methods

### Plant Materials and Growth Conditions

In total, 237 cultivated (*Hordeum vulgare*) and 190 wild (*Hordeum spontaneum*) barley originating from 28 countries (**Supplemental Table S1**) were used in this study to screen for drought tolerance. Seeds were surface-sterilized for 15 min with 10% commercial NaClO (0.52% final concentration of active Cl) and then rinsed for 30 min with tap water (Cai et al., 2019). Sterilized seeds were placed on BSM (basic salt media, 0.5 mM KCl and 0.1 mM CaCl_2_)-soaked filter paper in petri dishes and germinated in the dark at 25 ± 1°C for 48 h in a controlled growth chamber. Uniform well-germinated seeds were transplanted into 50-hole seedling trays (5 × 5 cm each hole) filled with vermiculite of equal volume. For each barley genotype, ten germinated seeds were planted in five holes in five individual trays with two seeds in each hole. All the trays were numbered and placed on 17 double-layered racks (L125 × W55 × H220 cm) in a well-controlled plant growth room (approx. 30 m^2^) with four trays on each layer. Barley seedlings were grown with one-fifth Hoagland solution (pH 6.0) (Wu et al., 2016) with a photoperiod of 16/8 h, light intensity of 200 ± 25 µmol·m^−2^·s^−1^, temperature of 22/18°C (day/night), and relative humidity of 60%. The nutrient solution in each tray was maintained at the same level by adding the solution twice per day with measuring cylinder. With barley seedlings growing to have two fully-expanded leaves (12 days post transplantation), the drought treatments were imposed.

### Drought Stress Treatments

#### Screening for Drought Tolerance

To investigate the genotypic variation in drought tolerance, 12-day barley seedlings were subjected to drought stress in two ways: 1) terminate the supply of nutrient solution for 10 days, termed as water deficit (WD); and 2) elevate the osmotic potential in trays by washing vermiculite with 20% (w/v) PEG8000 (P103734, Aladdin, Shanghai, China) solution (prepared in the background of one-fifth Hoagland solution. Tavakol et al., 2018; Cai et al., 2019) for three times and keep the osmotic potential with 20% (w/v) PEG8000 solution for another 5 days, termed as PEG-simulated drought (PEG). The seedlings grown with Hoagland solution under the same environmental condition were considered as control. The whole experiment was set up in a randomized block design with three treatments being three blocks. In each block, all the trays were swapped randomly every day to minimize the systematic error induced by spatial location. At the end of drought treatments, the shoots of barley seedlings were excised from basal conjunction to determine fresh weight, dry weight and water content. In the experiment of screening, fresh shoots were sampled from barley seedlings grown under either control or drought conditions and immediately weighed to obtain the shoot fresh weight (FW). Thereafter, the weighed shoots were dried in the oven at 75°C to constant weight and then weighed for the shoot dry weight (DW). The water content (WC) in shoot was calculated using the following formula: WC = (FW − DW)/FW × 100. Five replicates were determined for each combination of genotype and drought treatment.

#### Verification of Drought Tolerance

Based on the results from the work of screening for drought tolerance, eighteen barley genotypes, which displayed the consistent performance under both water-deficit and PEG-simulated drought stresses, were selected for further verification of their tolerance to drought stress. Barley seedlings were grown in 0.6 L pots containing vermiculite with nine plants per pot. Each barley genotype was grown in three individual pots. The growth condition and water-deficit drought treatment were the same as described above. The plants well-irrigated with adequate one-fifth Hoagland solution were considered as the control. The plants were grown for 10 d before drought stress started. After onset of water-deficit drought stress for 14 d, the seedlings were sampled for investigation of fresh weight, dry weight, water content, leaf chlorophyll content and fluorescence, leaf stomatal conductance and leaf sap osmolality. In the experiment of verification, shoot fresh and dry weights were determined as described for screening. To determine the relative water content (RWC) in barley leaves, the entire fresh oldest and youngest fully-expanded leaves from barley seedlings grown under control and drought conditions were respectively sampled and weighed immediately for the fresh weight (FW). The weighed leaves were then soaked in deionized water for 2 h at room temperature, then leaves were dried with kimwipes (34155, Kimtech, Kimberly-Clark, USA) softly and their turgid weights (TW) were recorded immediately. Relative water content was calculated using the following formula: RWC = (FW − DW)/(TW − DW) × 100. Five replicates were determined for each combination of genotype and drought treatment.

### Chlorophyll Content, Chlorophyll Fluorescence, and Stomatal Conductance

Chlorophyll content of both the oldest and youngest fully-expanded leaves was measured using a SPAD meter (SPAD-502 Plus, Konica Minolta, Inc. Tokyo, Japan). To measure leaf chlorophyll fluorescence, barley seedlings were dark-adapted for 30 min, and the chlorophyll fluorescence was measured for the oldest and youngest fully-expanded leaves using a portable fluorimeter (OS-30p+, Opti-Sciences, Inc. Hudson, NH, USA). The maximum quantum efficiency of photosystem II (*F_v/m_* = (*F_m_* − *F_o_*)/*F_m_*) was recorded at a saturating actinic light (660 nm) with an intensity of 1,100 µmol·m^−2^·s^−1^. Stomatal conductance (*gs*) was recorded from the youngest fully-expanded leaves using a porometer (Leaf Porometer SC-1, Decagon Devices, Inc. Pullman, WA, USA). All the above measurements were conducted in the middle part of the fully-expanded leaves following the manufacturer’s instructions. Five replicates were randomly taken for each barley genotype under either control or drought conditions.

### Leaf Sap Osmolality

Both the oldest and youngest fully-expanded leaves and stems were collected separately and stored in 1.5 ml centrifuge tubes immediately at −20°C. Tissue sap was extracted through freeze–thawing method. The extracts were centrifuged at 10,000 g for 3 min, and the supernatant was then collected in clean tubes. Osmolality was determined using a vapor pressure osmometer (Vapro 5600, Wescor Inc. Logan, UT, USA).

### Statistical Analysis

Statistical analyses were performed using SPSS Statistics 20 (IBM Corp. Armonk, New York, NY, USA). All data in the figures were given as mean ± SD. General Linear Model ANOVA analysis was used to confirm the significance of the means. Significance of differences were compared by Tukey HSD (honestly signiﬁcant difference) test. The regression between two drought treatments was fitted with SigmaPlot 12.5 (Systat Software, Inc. San Jose, USA). Cluster analysis of 427 genotypes based on shoot fresh weight, dry weight, and water content was conducted using TBtools (Chen et al., 2020). Hierarchical cluster analysis and Partial Least Squares-Discriminant Analysis (PLS-DA) on barley genotypes from the experiments of both screening and verification were conducted by MetaboAnalyst online analysis software 4.0 (Chong et al., 2019). Correlation analysis between screening and verification and between physiological parameters in the verification was performed with R script.

## Results

### Considerable Genotypic Variations in Drought Tolerance Exist for Both Cultivated and Wild Barley Under Two Different Drought Treatments

**Figure 1:**
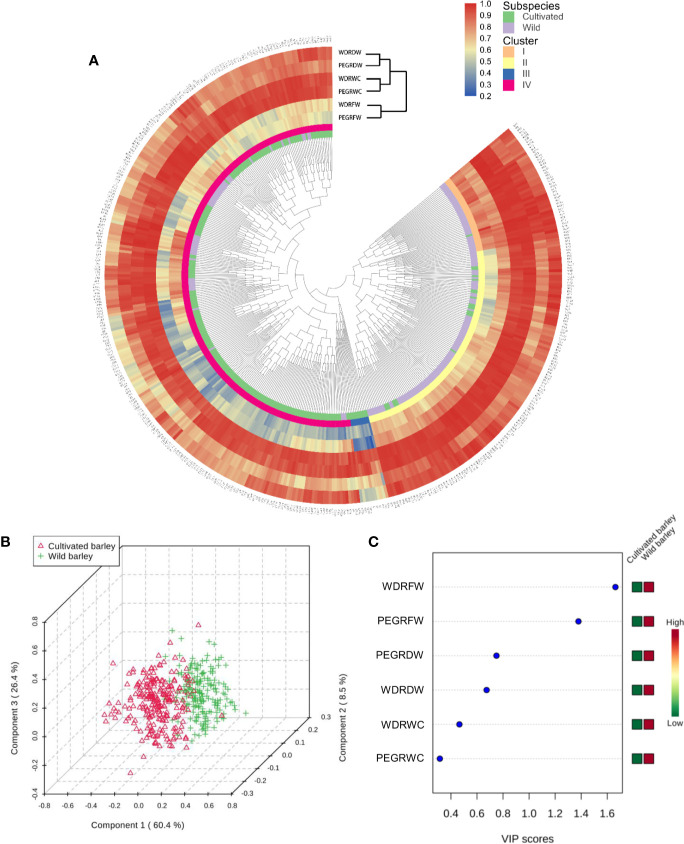
Classification of 237 cultivated and 190 wild barley genotypes according to relative shoot fresh weight, dry weight, and water content of drought to control. **(A)** Cluster analysis of 427 barley genotypes; **(B)** 3D scores plot between the selected principal components of PLS-DA; **(C)** Important features identiﬁed by PLS-DA. PEGRFW, PEGRDW, PEGRWC: Relative fresh weight, dry weight, and water content of drought to control under PEG-simulated drought; WDRFW, WDRDW, WDRWC: Relative fresh weight, dry weight, and water content of drought to control under water-limited drought.

Compared with control, both water deficit and PEG-simulated drought significantly reduced shoot fresh weight, dry weight, and water content in cultivated and wild barley, but a higher reduction was observed in cultivated than in wild barley (**Table 1**). The vermiculite was totally dried out at the end of experiments under water-limited drought conditions. It was easily found that both the drought treatments significantly inhibited the growth of barley seedlings (**Table 1**). Shoot fresh weight, dry weight, and water content were all decreased under drought treatments by 1.5–84.6%, 0.6–69.4%, and 0.1–37.5%, respectively (*P* < 0.01, **Table 1**). Considerable genotypic variation was observed either between the cultivated genotypes or between the wild ones under both control and drought conditions (*P* < 0.01, **Table 1**). The coefficient of variation among the cultivated and wild barley genotypes ranged from 0.17 to 0.35 for fresh weight, 0.16 to 0.57 for dry weight, and 0.02 to 0.07 for water content, respectively. Likewise, significant difference was observed between barley subspecies (*P* < 0.05, **Table 1**). Wild barley showed higher shoot fresh weight and dry weight than the cultivated barley under both WD and PEG conditions, suggesting a better performance of wild barley than cultivated barley under drought stress. However, the difference in shoot water content (ShootWC) between two barley subspecies was not significant (**Table 1**). Hierarchical cluster and Partial Least Squares-Discriminant Analysis (PLS-DA) was then conducted based on the reduction of shoot fresh weight, dry weight, and water content in all barley genotypes under both WD and PEG conditions (**Figure 1**). Results showed that all the barley genotypes used in this study were classified into four clusters, including 42 genotypes (one cultivated and 41 wild) in cluster I, 118 genotypes (20 cultivated and 98 wild) in cluster II, 10 genotypes (all cultivated) in cluster III, and 257 genotypes (206 cultivated and 51 wild) in cluster IV (**Supplemental Table S2**), with cluster I tending to be more tolerant to drought stress than the other clusters. Furthermore, most of cultivated and wild barley genotypes were clearly separated (**Figures 1B**). The most important features causing segregation of drought tolerance between barley genotypes were the reduction in fresh weight under WD and PEG conditions (**Figure 1C**).

**Table 1:** The performance of 237 cultivated and 190 wild barley genotypes under different drought treatments.

	PEG-simulated						Water-deficit						
	Cultivated barley			Wild barley			Cultivated barley			Wild barley			
	Control	Drought	Drought: Control	Control	Drought	Drought: Control	Control	Drought	Drought: Control	Control		Drought	Drought: Control
**Fresh weight (g·plant^−1^)**													
Min	0.36	0.12	0.22	0.40	0.17	0.24	0.54	0.26	0.15	0.60		0.22	0.18
Max	1.50	0.65	0.85	1.82	0.74	0.98	2.06	0.74	0.80	2.11		0.87	0.88
Mean	0.64	0.30	0.46	0.71	0.41	0.61	1.13	0.50	0.46	1.03		0.61	0.62
CV	0.29	0.35	0.25	0.35	0.21	0.24	0.22	0.17	0.25	0.23		0.17	0.24
Genotypes	**	**		**	**		**	**		**		**	
Drought treatments	**			**			**			**			
Subspecies	*						*						
Drought types	N.S.												
**Dry weight (g·plant^−1^)**													
Min	0.04	0.02	0.34	0.04	0.02	0.34	0.05	0.04	0.39	0.09		0.06	0.31
Max	0.24	0.13	0.95	0.28	0.16	0.99	0.32	0.16	0.98	0.33		0.17	1.00
Mean	0.08	0.05	0.72	0.09	0.07	0.78	0.14	0.10	0.74	0.15		0.11	0.79
CV	0.49	0.42	0.16	0.57	0.35	0.18	0.26	0.21	0.19	0.27		0.16	0.19
Genotypes	**	**		**	**		**	**		**		**	
Drought treatments	**			**			**			**			
Subspecies	*						*						
Drought types	N.S.												
**Water content (%)**													
Min	81	68	0.83	80	64	0.78	81	57	0.66		82	53	0.63
Max	92	90	0.98	92	90	1.00	92	90	0.99		89	86	0.99
Mean	88	81	0.92	88	84	0.95	88	79	0.90		85	81	0.95
CV	0.03	0.04	0.03	0.03	0.05	0.04	0.02	0.07	0.06		0.02	0.05	0.04
Genotypes	**	**		**	**		**	**			**	**	
Drought treatments	**			**			**				**		
Subspecies	N.S.						N.S.						
Drought types	N.S.												

*, **: Significance at P < 0.05 or P < 0.01 level. N.S., Not significant.

**Figure 2:**
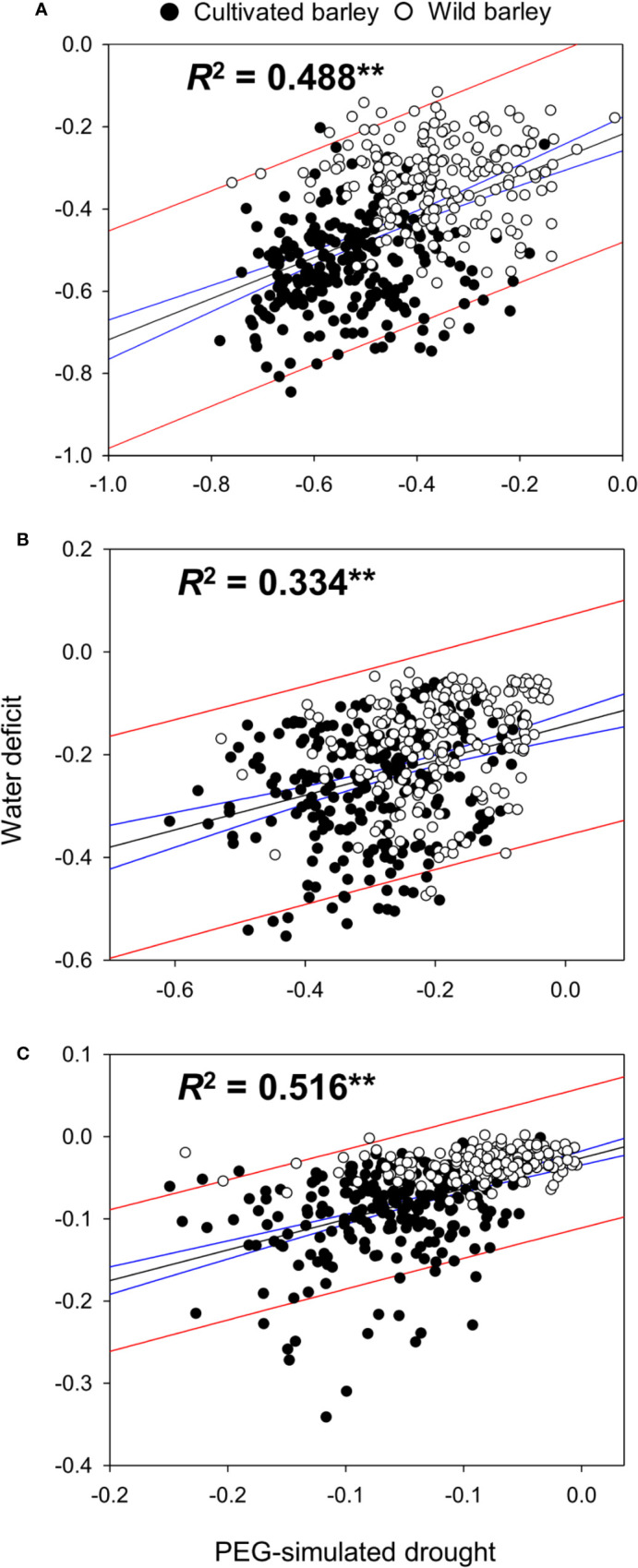
Correlation of the adverse impacts between PEG-simulated drought and water deficit. The correlation between two drought treatments was analyzed based on the relative fresh weight **(A)**, dry weight **(B)** and water content **(C)** in shoots of all 427 barley genotypes. ** indicates the significant difference at *P* < 0.01. Black line indicates the dynamic regression fit curve between two drought treatments, and blue and red lines indicate the 95% confidence and 95% prediction of the regression respectively.

The effect of drought type (*i.e.* WD and PEG) on the performance of barley seedlings under drought stress in shoot fresh weight, dry weight, and water content was also analyzed. Surprisingly, no significant difference between WD and PEG was observed for all the measurements (**Table 1**). A correlation analysis based on the reduction of shoot fresh weight, dry weight and water content in all barley genotypes was conducted between WD and PEG treatments. As expected, a very high correlation (r = 0.78, *P* < 0.0001, **Figure 2**) was found between the two drought treatments in this study, further proving the non-significant difference between WD and PEG.

### Verification of the Drought Tolerance of Barley Genotypes Under Water Deficit

**Figure 3:**
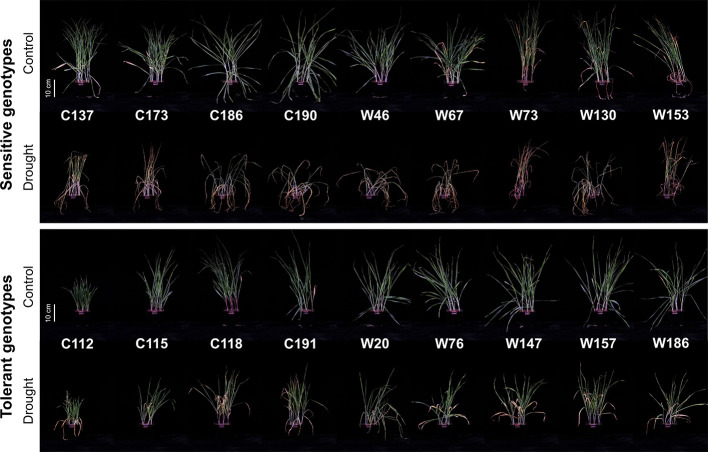
Performance of 18 selected barley genotypes under the control and water-limited drought conditions.

To verify the reliability of the drought tolerance screening results, eighteen genotypes with consistent performance under WD and PEG conditions were selected to investigate more measurements under the treatment of water deficit (**Supplemental Table S3**). Dramatic phenotypic difference was observed between drought sensitive and tolerant genotypes (**Figure 3**). After onset of 14-day water-deficit drought stress, the growth of shoot in drought sensitive genotypes was greatly inhibited and their leaves showed clear symptoms of wilting and chlorosis. Similar results were also found in the drought tolerant genotypes, but the symptoms of the inhibition in plant growth and leaf wilting were much lighter than the sensitive ones. Furthermore, the process of the growth development in drought-sensitive genotypes W73 and W130 was significantly delayed by the treatment of water deficit, whereas no such phenomenon was observed for the other genotypes. Next, the difference in drought tolerance between the sensitive and tolerant genotypes was further comparatively investigated with more measurements.

#### Biomass and Relative Water Content

**Figure 4:**
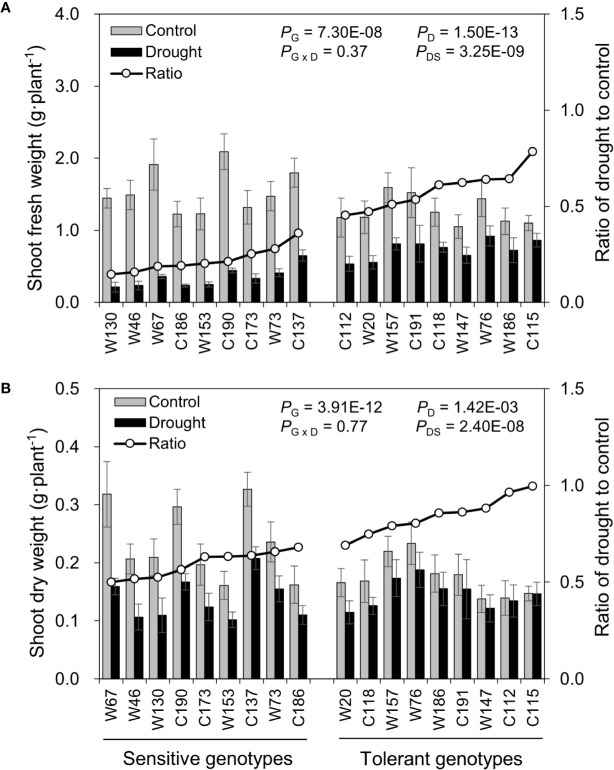
Effects of water-limited drought on shoot biomass production of 18 barley genotypes. **(A)** Shoot fresh weight; **(B)** Shoot dry weight. Data are mean ± SD of five individual measurements. G, genotype; D, drought treatment; G × D, interaction between genotype and drought treatment; DS, drought sensitivity.

**Figure 5:**
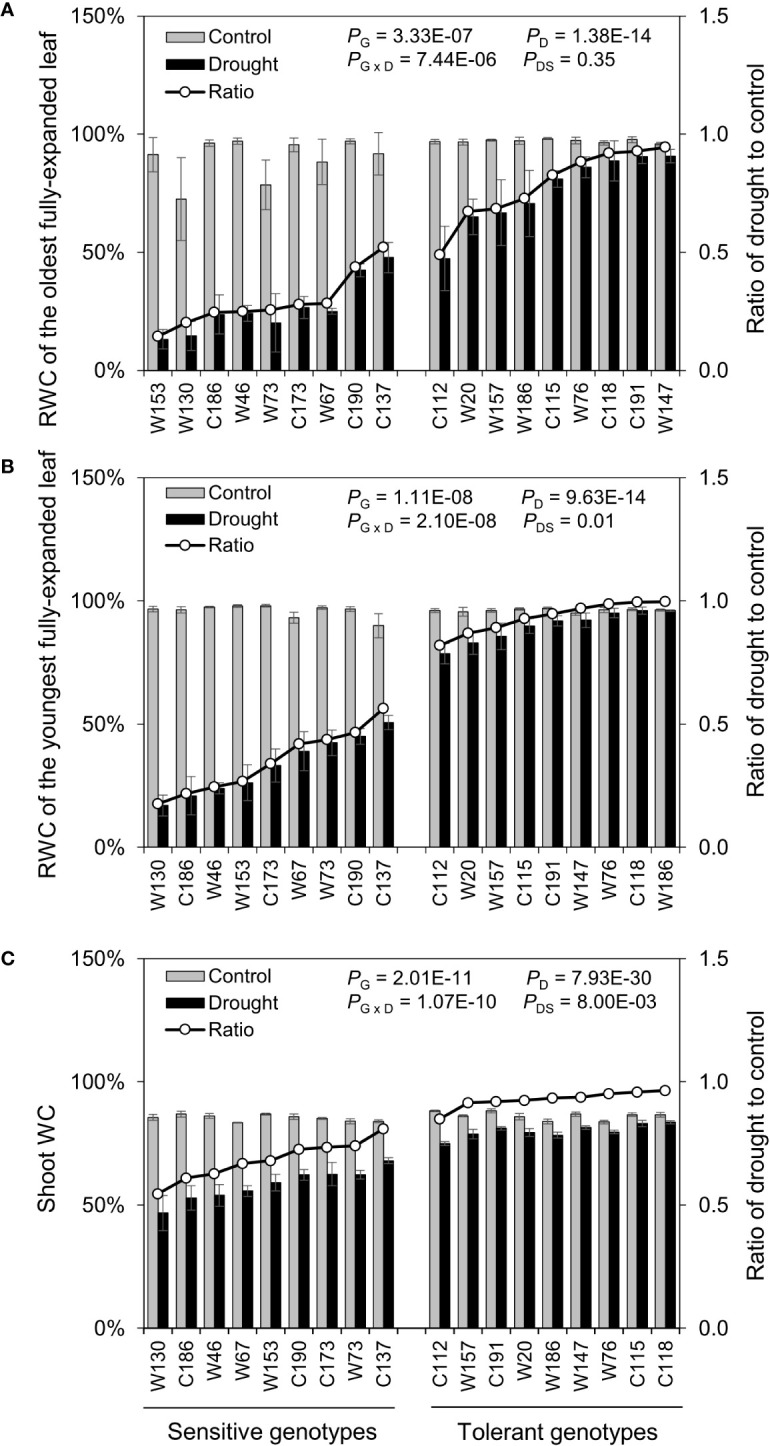
Effects of water-limited drought on the water relation of 18 barley genotypes. **(A, B)** Relative water content of the oldest and youngest fully-expanded leaves; **(C)** Water content of the whole shoot. Data are mean ± SD of five individual measurements. G, genotype; D, drought treatment; G × D, interaction between genotype and drought treatment; DS, drought sensitivity.

After the water-deficit drought treatment, five physiological parameters, including fresh weight, dry weight, and shoot water content and relative water content of the oldest (OLRWC) and youngest (YLRWC) fully-expanded leaves, were all reduced (**Figures 4** and **5**). To exhibit the difference between genotypes more clearly, the ratio of drought to control (Ratio_D:C_ = drought/control) was calculated for each genotype. The Ratios_D:C_ of shoot fresh weight in drought tolerant genotypes ranged from 0.45 to 0.78, significantly higher than those of drought sensitive genotypes ranging from 0.15 to 0.36 (**Figure 4A**). Likewise, the Ratios_D:C_ of shoot dry weight in tolerant genotypes (0.69–1.00) were nearly 2-fold of those in sensitive ones (0.50–0.68) (**Figure 4B**). The difference in leaf relative water content between sensitive and tolerant genotypes was much larger than that in shoot water content (**Figure 5**). On average, the tolerant genotypes displayed 2.7-fold higher Ratios_D:C_ in both OLRWC and YLRWC than the sensitive ones (**Figures 5A, B****)**. However, their difference in shoot water content was restricted to 1.4-fold (**Figure 5C**).

#### Chlorophyll Content, Chlorophyll Fluorescence and Stomatal Conductance

**Figure 6:**
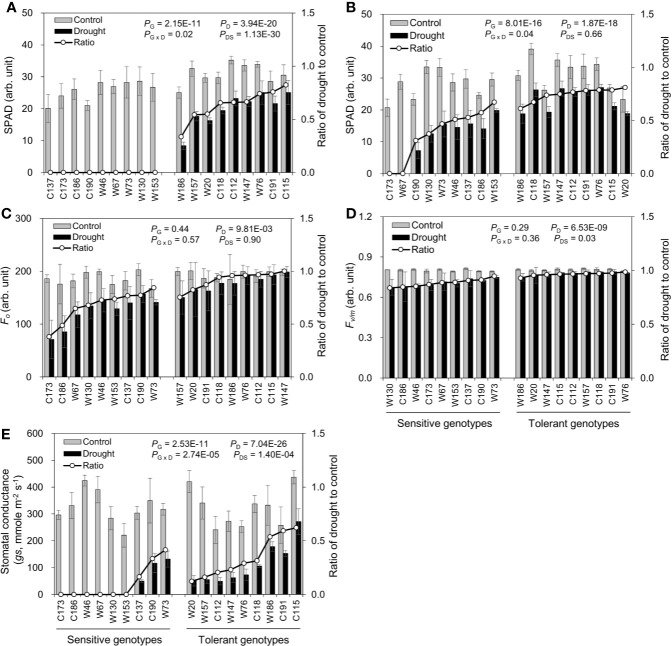
Effects of water-limited drought on chlorophyll content, chlorophyll fluorescence, and stomatal conductance of 18 barley genotypes. **(A, B)** SPAD value of the oldest and youngest fully-expanded leaf; **(C, D)** the initial (*F_o_*) and maximum quantum efficiency (*F_v/m_*) of photosystem II; **(E)** stomatal conductance (*gs*) of the youngest fully-expanded leaf. Data are mean ± SD of five individual measurements. G, genotype; D, drought treatment; G × D, interaction between genotype and drought treatment; DS, drought sensitivity.

Water-deficit drought stress dramatically decreased chlorophyll contents in both the oldest (OLSPAD) and youngest (YLSPAD) fully-expanded leaves, in a genotype-dependent manner (**Figures 6A, B****)**. Because of the total necrosis of the oldest leaves in drought-sensitive genotypes (**Figure 3**), their chlorophyll contents were all below the detectable level (**Figure 6A**). So, the chlorophyll fluorescence and stomatal conductance were no more measured in oldest fully-expanded leaf. On the other hand, OLSAPD values were all available in drought-tolerant genotypes with the Ratios_D:C_ ranging from 0.34 to 0.82 (**Figure 6A**). The symptom of chlorosis was much relieved in the youngest fully-expanded leaves, except the sensitive genotypes C173 and W67 (**Figure 6B**). Ratios_D:C_ of YLSPAD of drought-tolerant and -sensitive genotypes were 0.61–0.81 and N.D.–0.67, respectively (**Figure 6B**). Unexpectedly, the sensitive genotype W153 showed quite similar Ratios_D:C_ of YLSPAD with the tolerant genotypes W186 and C118, leading to the difference between the sensitive group and the tolerant one not significant. These results indicated that the chlorophyll content might not be a good screening proxy for drought tolerance in barley as reported in the previous studies (Hasanuzzaman et al., 2017; Banks, 2018). Initial chlorophyll fluorescence (YL*F*_o_), PSII photochemical efficiency (YL*F*_v/m_), and stomatal conductance (YL*gs*) in the youngest fully-expanded leaves were also measured (**Figures 6C–E**). Ratios_D:C_ of YL*F*_o_ among drought tolerant and sensitive genotypes were 0.75–1.00 and 0.38–0.84, respectively (**Figure 6C**). Drought-sensitive genotypes showed a very little difference in YL*F*_v/m_ with the tolerant ones, showing quite similar Ratios_D:C_ of YL*F*_v/m_ with them as 0.84–0.95 and 0.93–0.98, respectively (**Figure 6D**). YL*gs* was measured in only three drought-sensitive genotypes, with the Ratios_D:C_ ranging from 0.16 to 0.42 (**Figure 6E**). Ratios_D:C_ of YL*gs* in drought-tolerant genotypes varied from 0.12 to 0.62. Surprisingly, drought-sensitive C190 and W73 maintained relatively high Ratios_D:C_ of YL*gs* as some drought-tolerant genotypes like W76 and C118, suggesting an inappropriateness of *gs* as the selection criterion for drought tolerance.

#### Leaf and Stem Sap Osmolality

**Figure 7:**
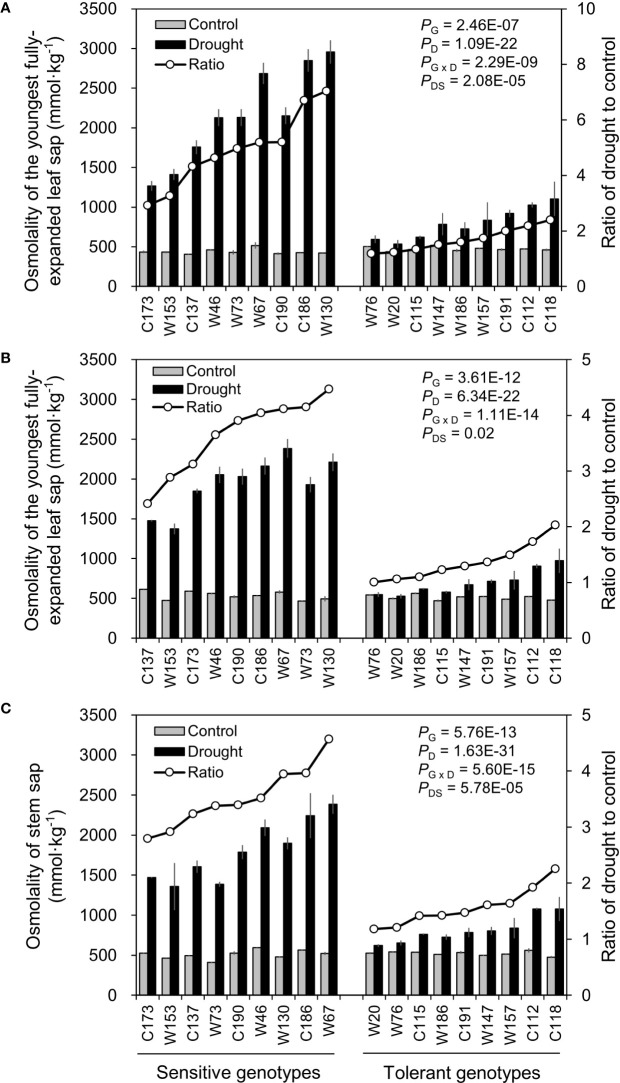
Effects of water-limited drought on tissue sap osmolality of 18 barley genotypes. **(A, B)** Sap osmolality of the oldest and youngest fully-expanded leaf; **(C)** Sap osmolality of stem. Data are mean ± SD of five individual measurements. G, genotype; D, drought treatment; G × D, interaction between genotype and drought treatment; DS, drought sensitivity.

Osmotic adjustment by accumulating more osmoticums like sugars, amino acids and K^+^ is one of the most important strategies for plants to cope with drought stress. As expected, water-deficit drought stress in this study significantly increased the osmolality in barley leaves and stems (**Figure 7**). And, the extents of such increase in osmolality differed greatly between sampling tissues and barley genotypes. The osmolality in the oldest fully-expanded leaf (OLOsmo) was notably higher than those in the youngest fully-expanded leaf (YLOsmo) and stem (StemOsmo) due to the larger loss of water in it (**Figures 3** and **7**). Furthermore, drought-sensitive genotypes exhibited much more remarkable increase in OLOsmo, YLOsmo, and StemOsmo in comparison to drought-tolerant genotypes. On average, Ratios_D:C_ of OLOsmo, YLOsmo, and StemOsmo in drought-sensitive genotypes were 2.8-, 2.3-, and 2.1-fold larger than those in drought-tolerant ones.

### The Reliability of the Screening for Drought Tolerance and the Promising Selection Criterion for It

**Figure 8:**
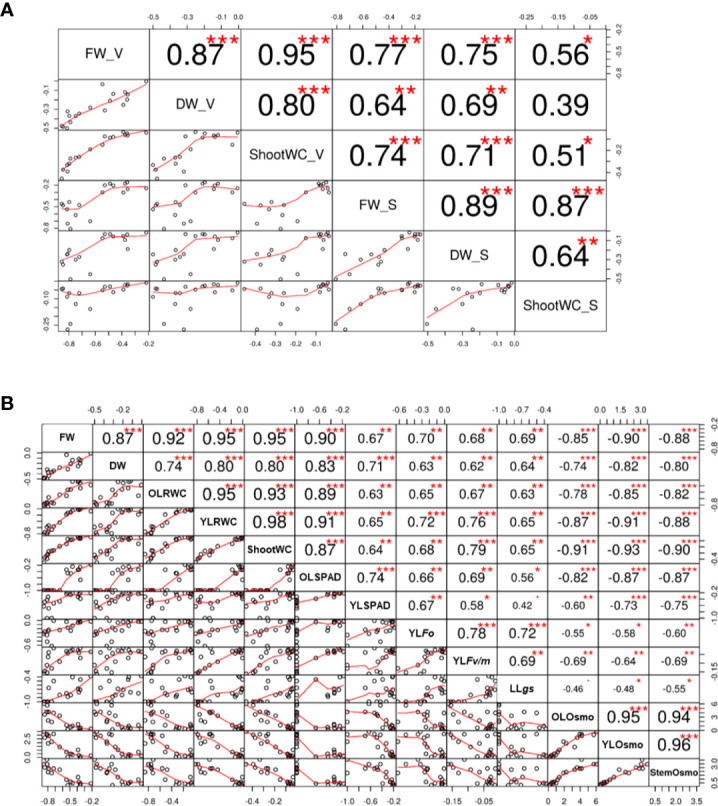
Correlation between physiological parameters. **(A)** Correlation of the data between the experiments of screening and verification of drought tolerance; **(B)** Correlation of physiological parameters in the experiment of drought tolerance verification. S, screening; V, verification; OL, the oldest fully-expanded leaf; YL, the youngest fully-expanded leaf; FW, fresh weight; DW, dry weight; RWC, relative water content; *gs*, stomatal conductance; Osmo, osmolality. *, ** and *** indicate the significant difference at *P* < 0.05, *P* < 0.01 and *P* < 0.001, respectively.

**Figure 9:**
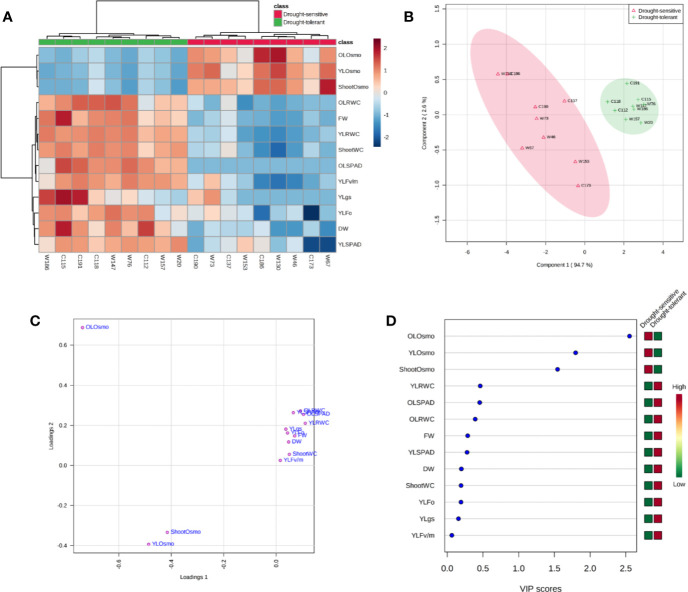
Classification of 13 physiological parameters in the experiment of drought tolerance verification. **(A)** Hierarchical cluster analysis; **(B**, **C)** 2D scores plot between the selected principal components and loadings plot for the selected principal components of PLS-DA; **(D)** Important features identiﬁed by PLS-DA. OL, the oldest fully-expanded leaf; YL, the youngest fully-expanded leaf; FW, fresh weight; DW, dry weight; RWC, relative water content; *gs*, stomatal conductance; Osmo, osmolality.

Correlation analysis based on the traits of fresh weight, dry weight, and shoot water content revealed a high consistence between the experiment of screening and the one of verification (**Figure 8A**), and the results of hierarchical cluster analysis and principle component analysis (PCA) also showed that the sensitive and tolerant genotypes were clearly separated (**Figures 9A, B****)**. These results demonstrated the reliability of the practice of screening for drought tolerance in this study. Next, the correlation of four categories of physiological parameters in the experiment of verification, including biomass (FW and DW), water relation (OLRWC, YLRWC, and ShootWC), photosynthetic activity (OLSPAD, YLSPAD, YL*F_o_*, YL*F_v/m_*, and YL*gs*), and osmotic adjustment (OLOsmo, YLOsmo, and StemOsmo), were further analyzed to certify the drought tolerance of the selected barley genotypes (**Figure 8B**). Results showed that biomass of barley seedlings remarkably correlated with the parameters of water relation (r = 0.74–0.95, *P* < 0.001), photosynthetic activity (r = 0.62–0.90, *P* < 0.01), and osmotic adjustment (r = −0.74–−0.90, *P* < 0.001), and the correlation of fresh weight with these parameters were all comparatively higher than dry weight (**Figure 8B**). Furthermore, water relation strongly correlated with the process of osmotic adjustment as expected (r = −0.78–−0.93, *P* < 0.001; **Figure 8B**). However, much lower correlation was observed between photosynthetic activity and the other three categories of parameters (**Figure 8B**). The hierarchical cluster and PLS-DA analysis of these parameters indicated that the traits of osmotic adjustment differed greatly with the other traits involved in biomass production, water relation, and photosynthetic activity (**Figures 9A, C****)**. A further analysis of the Variable Importance in Projection (VIP) showed that OLOsmo, YLOsmo, and StemOsmo were the most important PLS loadings to explain the genotypic variation in drought tolerance, and their best alterative was YLRWC (**Figure 9D**).

## Discussion

### Natural Populations With Wide Diversity Are the Valuable Source for Evaluating Drought Tolerance in Barley

Identifying and selecting the true drought-tolerant genotypes is very challenging. The key point of identifying the true drought-tolerant genotypes is the reliable phenotyping. In the past decades, the assessment of drought tolerance has been widely conducted in the drought tolerant crop breeding programs (Labuschagne et al., 2008; Jain et al., 2013; Monneveux et al., 2013; Djemel et al., 2018). To accelerate the identiﬁcation of useful parents and genes, most of these researches used the bi-parental populations from the genetically dissimilar parents as the phenotyping plant materials. However, the genetic diversity in barley has been drastically narrowed during long-term domestication, especially by the activities of modern breeding and cultivation (Nevo and Chen, 2010). As a result, the chance of identifying the useful genes conferring drought tolerance has been sharply restricted, consequently leading to very limited success has been obtained in the drought tolerant crop breeding (Cominelli et al., 2013; Fita et al., 2015). Various studies on the breeding for drought tolerance have suggested the importance of diverse natural populations in the identification of useful parents and genes for improving drought tolerance in crops (Varshney et al., 2012; Jabbari et al., 2018; Sallam et al., 2019). In the present study, 237 cultivated and 190 wild barley genotypes originating from 28 countries were screened for drought tolerance. These barley genotypes existed with great variation in shoot biomass (fresh and dry weight) and water content under both control and two drought conditions (water deficit and PEG-simulated) (**Table 1**). Moreover, two barley subspecies, cultivated and wild, significantly differed from each other in drought tolerance, with better performance observed in wild barley than cultivated barley under drought stress (**Table 1**; **Figure 1**). Similar results have been reported in the previous studies on Tibet wild barley (Zhao et al., 2010) and Fertile Crescent wild barley (Tyagi et al., 2011). According to hierarchical cluster analysis, all the barley genotypes could be classified into four clusters (**Figure 1A**). We next selected 18 genotypes to verify the reliability of the screening with more investigations. As expected, the selected drought-sensitive and -tolerant genotypes exhibited the consistent difference in performance under drought condition as they did in the experiment of screening, and could be significantly distinguished with each other (**Figures 3****–****7**, **9**). These genotypes displayed a great potential as the contrasting parents to generate the bi-parental populations for identifying the elite QTLs and genes conferring drought tolerance. All these results suggest that the 427 barley genotypes used in this study, especially the wild barley, could be a valuable plant material source for identifying useful parents and genes in drought tolerance breeding.

### Evaluating the Same Materials Under Different Drought Conditions Is More Promising to Identify the True Drought Tolerant Genotypes

In general, drought is a slow-onset and intricate stress to plants, and the severity of drought depends on various factors, like amount of watering, duration of water deficit, irrigation method, soil properties, and environmental temperature (Macnicol et al., 1993; Savin and Nicolas, 1996; Wallwork et al., 1998). Many studies have observed the spatial and time variation in drought tolerance assessment because the drought stress usually is hard to be repeated between locations and years (Zekri, 1991; Raggi, 1992). Therefore, it is very necessary to test the drought tolerance in more than one location or/and year in the target environments, or even better under controlled conditions (Sallam et al., 2018). So far, a lot of efforts have been given to develop the uniform, stable, and reliable drought condition. Pot culture with soil seems a good attempt to provide plants the most similar environments as field conditions. However, soil is complicated in constituents, and it is very difficult to maintain a uniform and constant water status and nutrient availability (Passioura, 2006; Munns et al., 2010), resulting in a reduction in the repeatability in drought severity and consequently the efficiency of phenotyping. Another successful attempt is elevating the osmotic potential in the external environment to simulate the drought stress with PEG (Kaufmann and Eckard, 1971). PEG has a high molecular weight (6,000 or 8,000 g·mol^−1^), and it is is easy to induce the satisfied and constant osmotic stress in the culture using a timescale of days (Lawlor, 1970; Szira et al., 2008). It has been previously reported that drought stress induced by PEG had similar effects on the growth parameters as did by water withholding (Molnár et al., 2004), and it generated reproducible effects on plant growth at seedling stages (Szira et al., 2008). Thus, PEG solutions with different concentrations have been widely applied in studies on evaluating and phenotyping of drought tolerance (Szira et al., 2008; Munns et al., 2010; Luo et al., 2015; Meng et al., 2016). However, there is a consideration that osmotic stress is too simple and quick-effect to delegate the complicated and slow-onset drought stress (Dubois and Inzé, 2020). Therefore, it is preferable and recommendable to evaluate the same genotypes under different drought conditions. In the present study, the phenotyping of drought tolerance in barley genotypes was conducted under the conditions of both water-deficit and PEG-simulated drought. Although the two types of drought treatments spent different times before the symptoms of stress appeared, there was no significant difference observed between water-deficit and PEG-simulated drought in the inhibition effects on barley plants (**Table 1**). Furthermore, their adverse impacts on barley growth were significantly positively correlated when fresh weight, dry weight, and water content of shoots were all taken into consideration (**Figure 2**). All these results revealed the reliability of the screening for drought tolerance under water-deficit and PEG-simulated drought conditions in this study, which was further demonstrated by further investigations of 18 genotypes (**Figures 3****–****9**).

Growth medium is another noteworthy factor affecting the practice of screening. PEG is easy to make osmotic solutions at different concentrations, but PEG solutions are viscous and the viscosity increases as concentrations increase, so O_2_ diffusion to roots is inevitably limited at varying levels and hypoxia stress may occur (Mexal et al., 1975; Verslues et al., 1998; Munns et al., 2010). In addition, without supporting in solutions, roots are prone to be damaged during aeration and changing solutions (Miller, 1987), which may increase the penetration of PEG into plants and consequently affect their performance in response to drought stress. In this study, we used vermiculite, which is odorless, lightweight, non-toxic, and sterile and will not rot, deteriorate or mold, as the growth media to support barley roots. In this way, growth medium had a uniform composition, and gas permeability and water retention were well balanced. It not only reduced the occurrence of the other stimuli than osmotic stress to affect the accuracy of the experiment, but also was time- and labor-saving and suitable for large-scale screening practice.

### Using the Appropriate Assay and Traits Is Critical to Obtain the Accurate Phenotyping for Drought Tolerance

The assessment of drought tolerance is the prerequisite of all studies concerning drought tolerance (Cattivelli et al., 2008). Drought tolerance is determined by identifying a trait that can be used to measure the effects of drought stress on plants (Sallam et al., 2019). Traditionally, drought tolerance is usually estimated using yield loss under drought conditions, but field trials are time-, cost- and labor-consuming and easily affected by environmental variation (Yadav et al., 2019). So, increasing studies are on phenotyping drought tolerance using other traits tightly related to drought tolerance, including morphology (root and plant), biomass production (leaf and shoot fresh weight and dry weight), water relation (relative water content and leaf wilting), photosynthetic activity (SPAD, *F_v/m_*, *gs*, *An*, *etc*.), osmotic adjustment (Osmo, accumulation of amino acids, sugars, polyols *etc*.), nutrient relations (K^+^, Ca^2+^, *etc*.), and oxidative status (enzymatic activity, ROS accumulation, MDA) (Lawlor and Cornic, 2002; Oukarroum et al., 2007; Szira et al., 2008; Hargreaves et al., 2009; Munns et al., 2010; Zhao et al., 2010; Cai et al., 2019; Yadav et al., 2019). Of them, fresh matter, dry matter, and relative water content are basic traits that are widely used in drought experiments in barley (Sallam et al., 2019). Accordingly, the reduction in shoot fresh weight, dry weight, and water content was used as the traits for the screening practice of drought stress in the present study (**Table 1**; **Figures 1** and **2**). Indeed, no matter under what kind of drought conditions, the contrasting genotypes in drought tolerance were successfully identified using these three traits (**Figure 3**), which was verified by the other traits (**Figures 4****–****7**). However, the investigation of these traits is a destructive method, which does not allow the “outstanding” individuals to be tracked in long term and be later used in a drought tolerance breeding program. So, the other simple, rapid, and nondestructive methods are required for the large-scale screening of drought tolerance. In this case, 18 contrasting barley genotypes identified in the experiment of screening were further investigated in terms of biomass production (shoot fresh weight and dry weight), water relation (OLRWC, YLRWC, and ShootWC), photosynthetic activity (OLSPAD, YLSPAD, YL*F_o_*, YL*F_v/m_*, and YL*gs*), and osmotic adjustment (OLOsmo, YLOsmo, and StemOsmo) to find out the feasible traits as screening criteria of drought tolerance in barley at seedling stage. As expected, the selected drought-tolerant genotypes showed much less reduction in shoot fresh weight and dry weight than the drought-sensitive ones under water-deficit drought condition (**Figure 4**), which was significantly positively correlated with the results of screening (**Figure 8A**). Likewise, the contrasting genotypes differed greatly in water relation (YLRWC and ShootWC), photosynthetic activity (OLSPAD, YL*F_v/m_*, and YL*gs*), and osmotic adjustment (OLOsmo, YLOsmo, and StemOsmo) under the water-limited drought stress (**Figures 5****–****7**). Furthermore, all these investigated traits were not only significantly correlated with each other (*P* < 0.05–0.001) but also remarkably correlated with biomass (**Figure 8B**), indicating that any of them has the potential to be the selection criterion for drought tolerance screening. Indeed, fresh matter, dry matter, and relative water content have been widely used in drought experiments in barley (Sallam et al., 2019). In chickpea, a significant and well-defined relationship was also observed between relative water content and drought tolerance (Gunes et al., 2008). In addition, chlorophyll fluorescence has been recommended as a tool to identify drought stress in *Acer* species and barley (Hasanuzzaman et al., 2017; Banks, 2018). In the present study, surprisingly, the traits of osmotic adjustment (OLOsmo, YLOsmo, and StemOsmo) were assessed as the most important features to explain the genotypic variation in drought tolerance by PLS-DA analysis (**Figure 9D**). Unfortunately, the measurement of osmolality on stem is also destructive, which dramatically diminished the practical value of its utilization in screening for tolerance. On the other hand, it should be noted that the necrosis and wilting of the oldest fully-expanded leaf in some genotypes may be attributed to physiological senescence, as they were also visualized even under control condition (**Figure 3**). Therefore, the measurements on the oldest fully-expanded leaf could not be recommended as the credible traits for drought tolerance in barley. Accordingly, only the measurement of osmolality on the youngest fully-expanded leaf (YLOsmo) was left. However, single-trait evaluation for drought tolerance to distinguish between tolerant and sensitive genotypes is quite risky and is normally not recommendable. To address this issue, the relative water content in the youngest fully expanded leaf (YLRWC), the VIP score of which ranked right behind the traits of osmotic adjustment, is the best alternative of credible trait for drought tolerance as YLOsmo. Actually, it has been already reported that relative water content in the youngest fully-expanded leaf of cowpea, mungbean, and snap bean estimated during pod formation and in flag leaf of wheat during anthesis was positively correlated with seed yield (Kumar and Sharma, 2010). In addition, the measurements of osmolality and relative water content in leaf can be easily conducted using several leaf discs and cause little damage to plants. Taken together, sap osmolality and relative water content in the youngest fully-expanded leaf are the appropriate selection criteria of screening for drought tolerance in barley at seedling stage.

## Conclusions and Prospects

Both the cultivated and wild barley subspecies displayed the considerable genotypic variation in drought tolerance, showing a potential value of these barley germplasms for identifying the useful parents and genes for drought tolerance breeding. Although no significant difference was found between PEG-simulated drought and water-deficit drought in their adverse effects on barley seedlings, it is more promising to identify the true and stable drought tolerant genotypes under two drought conditions. Sap osmolality and relative water content in the youngest fully-expanded leaf could be the highly suitable selection criteria of screening for drought tolerance in barley at seedling stage.

## Data Availability Statement

The raw data supporting the conclusions of this article will be made available by the authors, without undue reservation.

## Author Contributions

KC, GZ, and FZ conceived and designed the research. KC and FZ conducted the experiments. KC and FZ wrote the manuscript. All authors contributed to the article and approved the submitted version.

## Funding

This work was supported by the National Natural Science Foundation of China (grant Numbers: 31371559 and 31571599) to FZ, and Jiangsu Collaborative Innovation Center for Modern Crop Production (JCIC-MCP).

## Conflict of Interest

The authors declare that the research was conducted in the absence of any commercial or financial relationships that could be construed as a potential conflict of interest.

The handling editor declared a past co-authorship with several of the authors FZ, GZ.

## Supplementary Material

Supplemental Table S1 Barley accessions used for screening of drought tolerance in this study.

Supplemental Table S2 Detailed information of barley genotypes in four clusters.

Supplemental Table S3 Performance of the selected 18 barley genotypes in the experiment of screening.
